# The use of mechanical restraint and seclusion in patients with schizophrenia: A comparison of the practice in Germany and Switzerland

**DOI:** 10.1186/1745-0179-3-1

**Published:** 2007-02-04

**Authors:** Veronika Martin, Renate Bernhardsgrütter, Rita Goebel, Tilman Steinert

**Affiliations:** 1 Psychiatric Hospital „Weissenau", Dept. Psychiatry I of the University of Ulm, Ravensburg, Germany; 2 Psychiatric Hospital Wil, Wil, Switzerland

## Abstract

**Background:**

The use of coercive measures is an indicator of the quality of psychiatric inpatient treatment. To date, there is no data available to European comparisons on the incidence of such measures.

**Methods:**

The frequency and duration of mechanical restraint and seclusion on patients with a diagnosis of F2 ICD-10 was analysed in seven German and seven Swiss psychiatric hospitals in the year 2004 using three indicators. Differences between German and Swiss hospitals regarding the indicators were tested for statistical significance using Mann-Whitney-U-tests.

**Results:**

6.6 % (Switzerland) and 10.4 % (Germany) of admissions respectively were affected by mechanical restraint and 17.8 % (Switzerland) and 7.8 % (Germany) respectively by seclusion. Seclusion as well as mechanical restraint per case were applied significantly more often in German than in Swiss hospitals and were of significantly longer duration in Swiss than in German hospitals.

**Conclusion:**

The results showed different patterns in the use of seclusion and mechanical restraint across Swiss and German hospitals. For future European research on the use of compulsory measures in routine psychiatric care, there is a need for uniformed definitions, reliable documentation of coercive measures as well as for an identical way of data analysis. To meet these conditions is the first step to achieve European standards for the use of coercive measures.

## Background

Across Europe, restraint and seclusion are common interventions in psychiatric in-patient settings to deal with aggressive patient behaviour [1]. Since the 1990s there is a growing interest in the incidence of coercive measures in most European countries [2,3]. During the last years, there have been a few European studies, in which psychiatric hospitals were compared regarding the frequency and duration of coercive measures. These studies are from the UK [4], Switzerland [5], Finland [6] and Germany [7,8]. To date, there is no available data for European comparisons on the incidence of such measures. This is partly due to diversity of the following factors: the key indicators used, analysing data on patients versus cases, the type of coercive measure that is analysed and the mix of patient characteristics.

In Switzerland and Germany two independent working groups exist that deal with the prevention of violence and coercion in psychiatry. The German working group "Arbeitskreis zur Prävention von Zwang und Gewalt in der Psychiatrie" (Project of prevention of violence and coercion in psychiatry) [9] consists of 21 hospitals, located primarily in the South of Germany. The Swiss working group "Qualitätszirkel Benchmarking Zwangsmaßnahmen" (Benchmarking coercion in psychiatry) consists of 7 hospitals, located in the North-Eastern, German speaking part of Switzerland. Both working groups focus on a reduction of the frequency and duration of coercive measures and critically reflect on the clinical practice of the use of coercive measures. A cooperation between the two working groups was established in 2004.

Because of possible physical and psychological damage on patients affected by coercive measures [10], the use of coercive measures can be seen as an indicator of the quality of psychiatric inpatient treatment. A previous study in Germany yielded evidence, that except for demented patients who are mostly restrained for the prevention of falls, the patients mostly affected by compulsory measures in psychiatric hospitals are those with schizophrenic disorders [8]. The objective of the cooperation between the two working groups was to compare the use of coercive measures in patients with schizophrenic disorders in Germany and Switzerland.

## Methods

The use of mechanical restraint and seclusion on patients with an F2 ICD-10-diagnosis was analysed in seven German and seven Swiss psychiatric hospitals for the year 2004. The participating Swiss hospitals were the following:

- Clinic of Psychiatry Königsfelden

- Clinic of Psychiatry Luzern

- Clinic of Psychiatry Oberwil

- Clinic of Psychiatry Pfäfers

- Clinic of Psychiatry Solothurn

- Clinic of Psychiatry St. Urban

- Clinic of Psychiatry Wil

The participating German hospitals were the following:

- Clinic of Psychiatry Bad Schussenried

- Clinic of Psychiatry Calw

- Clinic of Psychiatry Emmendingen

- Clinic of Psychiatry Reichenau

- Clinic of Psychiatry Rottweil

- Clinic of Psychiatry Weissenau

- Clinic of Psychiatry Winnenden

### Hospital characteristics

All hospitals are completely responsible for psychiatric inpatient care in their defined catchment areas and supply full psychiatric treatment. In the Swiss hospitals the number of hospital beds varied between 53 and 242, the number of hospital beds in the German hospitals varied between 342 and 755. The number of hospital beds per 1000 inhabitants in the defined catchment areas varied between 0.3 and 0.8 for the Swiss and between 0.3 and 0.7 for the German hospitals.

In the Swiss hospitals 7931 cases were treated in 2004 of which 1976 (24.9%) had a schizophrenic disorder (F2). In the German hospitals 31399 cases were treated in 2004 of which 6761 cases had a schizophrenic disorder (21.5%). Diagnoses were made in clinical routine procedures according to ICD-10 criteria.

### Definitions

In all participating hospitals, seclusion and mechanical restraint were defined in an identical way. Mechanical restraint was defined as the use of belts to fix a patient to a bed. Seclusion was defined as bringing the patient into an empty and locked room without possibility to leave.

### Data recording

In all participating hospitals there are legal or administrational regulations which advice the documentation of seclusion and restraint. The documentation of coercive measures in both countries is looked upon as a legal requirement and staff's accuracy in the documentation of such procedures is generally very high.

Within the German as well as within the Swiss working group a documentation form for data on coercive measures was developed. The Swiss as well as the German documentation form comprises information on the patient's ICD-10 principal diagnosis (F0–F9), the code of the respective ward, the kind of coercive measure (mechanical restraint, seclusion, forced medication) and data on the beginning and end of the coercive measure. In addition, the Swiss documentation form comprises information on the use of force and the way of administration of forced medication (oral versus injection). The German documentation form additionally comprises gender, date of birth, date of admission and the patient's legal status as well as indication and legal basis for the applied coercive measure [11].

The use of the respective documentation form was implemented into clinical routine procedures. In five out of seven German and in four out of seven Swiss hospitals the documentation form was integrated into the electronic case note system of the hospital. Staff on the ward recorded the data locally. In the remaining two German and three Swiss hospitals paper-pencil versions of the documentation forms were used. The data was then centrally collected and manually recorded into an Access-mask of the respective German or Swiss documentation form. Since 2002 for the German and 2003 for the Swiss hospitals, the incidence and duration of coercive measures for patients with an ICD-10 F1–F9 diagnosis have been documented reliably and completely.

### Data analysis

For all participating hospitals the use of mechanical restraint and seclusion among patients with an F2 ICD-10 diagnosis was analysed for the one-year period of 2004. To analyse the data of the German hospitals, an Access programme called "Documentation of Coercive measures in Psychiatry" (DoComP) was generated specifically for this purpose. This programme evaluates hospital-based indicators in the respective ICD-10 principal group (F0–F9). Due to prescriptions of data protection in Germany the data of patients exposed to coercive measures was recorded anonymously and did only include information on the patients' date of birth, gender, date of admission and ICD-10 diagnosis (F0–F9). The programme identifies two records of coercive measures as belonging to the same case, if date of birth, gender and date of admission are identical.

According to former analysis [8], the following indicators were used for the comparison:

1. Percentage of cases exposed to mechanical restraint (or seclusion respectively):

Number of cases exposed to mechanical restraint (or seclusion respectively) divided by the number of cases treated. The quotient is then multiplied with a factor of 100.

2. Number of mechanical restraints (or seclusion respectively) per affected case:

Total number of mechanical restraints (or seclusion respectively) divided by the total number of cases affected by mechanical restraint (or seclusion respectively)

3. Mean duration of one mechanical restraint (or one seclusion respectively):

Total duration of mechanical restraints divided by total number of mechanical restraints (or seclusion respectively).

The indicators are related to cases treated i.e. admissions (no. 1) and cases affected by coercive measures (no.2 and no.3), respectively.

### Statistical methods

Differences between German and Swiss hospitals regarding the indicators were tested for statistical significance using Mann-Whitney-U-tests.

## Results

The tables 1, 2, 3, 4 present the number of interventions, the key data with mean and median as well as maximum and minimum values among the hospitals respectively. In one Swiss hospital mechanical restraint was not used.

**Table 1:** Number and proportion of mechanical restraint and seclusion in seven German and seven Swiss psychiatric hospitals

Interventions				
	**Germany**		**Switzerland**	

	mechanical restraint (number of hospitals = 7)	seclusion (number of hospitals = 7)	mechanical restraint (number of hospitals = 6)	seclusion (number of hospitals = 7)
N	2499	2510	168	643
%	49.9 %	50.1 %	20.7 %	79.3 %

In one Swiss hospital no mechanical restraint was used.

**Table 2:** Proportion of cases affected by mechanical restraint and seclusion in seven German and seven Swiss psychiatric hospitals

Proportion of affected cases				
	**Mechanical restraint**		**Seclusion**	

	Germany (number of hospitals = 7)	Switzerland (number of hospitals = 6)	Germany (number of hospitals = 7)	Switzerland (number of hospitals = 7)
cases affected	700 out of 6761 admissions	130 out of 1976 admissions	524 out of 6761 admissions	351 out of 1976 admissions
proportion	10.4 %	6.6 %	7.8 %	17.8 %*
minimum	7.3 %	1.0 %	0.4 %	8.2 %
maximum	15.9 %	14.4 %	14.3 %	38.3 %

In one Swiss hospital no mechanical restraint was used.

*: p < .05

**Table 3:** Number of mechanical restraint and seclusion per affected case in seven German and seven Swiss psychiatric hospitals

Number of interventions per affected case				
	**Mechanical restraint**		**Seclusion**	

	Germany (number of hospitals = 7)	Switzerland (number of hospitals = 6)	Germany (number of hospitals = 7)	Switzerland (number of hospitals = 7)
mean	3.4	1.5	4.2	1.6
median	3.2*	1.2	4.0*	1.4
minimum	2.6	1.0	2.4	1.0
maximum	4.6	3.0	7.5	2.7

In one Swiss hospital no mechanical restraint was used.

*: p < .05

**Table 4:** Average duration of any one mechanical restraint and any bone seclusion in seven German and seven Swiss psychiatric hospitals (in hours)

Average duration (hours)				
	**Mechanical restraint**		**Seclusion**	

	Germany (number of hospitals = 7)	Switzerland (number of hospitals = 6)	Germany (number of hospitals = 7)	Switzerland (number of hospitals = 7)
mean	9.6	48.7	7.4	55.0
median	9.3	52.8*	9.0	55.1*
minimum	6.2	10.4	4.1	21.1
maximum	14.8	75.6	11.0	87.5

In one Swiss hospital no mechanical restraint was used.

*: p < .05

A significantly higher percentage of cases was affected by seclusion in the Swiss than in the German hospitals (Mann-Whitney-U-Test, p < .05, N = 14). Seclusions (Mann-Whitney-U-Test, p < .05, N = 14) as well as mechanical restraints (Mann-Whitney-U-Test, p < .05, N = 13) on each affected case were applied significantly more often in German than in Swiss hospitals. Seclusions (Mann-Whitney-U-Test, p < .05, N = 14) as well as mechanical restraints (Mann-Whitney-U-Test, p < .05, N = 13) were of significantly longer duration in Swiss than in German hospitals.

In the Swiss hospitals 28 cases out of 1976 admissions (1.4 %) were affected by seclusion as well as by mechanical restraint and 453 out of 1976 cases treated (22.9 %) were exposed to at least one kind of intervention (mechanical restraint or seclusion). In the German hospitals 131 cases out of 6761 admissions (1.9 %) were affected by seclusion as well as by mechanical restraint and 1093 out of 6761 cases treated were exposed to at least one kind of intervention (16.2 %).

## Discussion

The results presented here provide epidemiological data on the use of mechanical restraint and seclusion in psychiatric inpatient care for patients with schizophrenic disorders in seven German and seven Swiss hospitals. For the first time, a comparison concerning the frequency and duration of seclusion and restraint in psychiatric hospitals across two countries was realised. Due to different definitions, the use of forced medication could not be considered in our data analyses.

The results clearly showed different patterns in the use of seclusion and mechanical restraint across Swiss and German hospitals. In German hospitals more cases were exposed to mechanical restraint than to seclusion, whereas in Swiss hospitals more cases were secluded than restrained. Restraints as well as seclusions per case were on average applied about three times more often in German than in Swiss hospitals. However, the duration of any one seclusion and of any one restraint was on average about five times higher in Swiss hospitals compared to the average within German hospitals. The variance within the hospitals of one country and between the countries can generally be considered as high. The different patterns in the use of seclusion and mechanical restraint might reflect different national traditions in the clinical practice. It seems that compared to Swiss staff, German psychiatric staff strive to keep the time of one coercive measure as short as possible. This conclusion can be drawn from the higher number of seclusions and mechanical restraints per affected case but the shorter average duration of any one seclusion and any one mechanical restraint in the German in comparison to the Swiss hospitals. The much higher average duration of any one seclusion and any one mechanical restraint in the Swiss compared to the German hospitals raises the question, whether in the Swiss hospitals seclusion and mechanical restraint were maintained longer than would have been clinically necessary. However, we were not able to relate our data to the number of events of violent patient behaviour, closed versus open ward, staffing levels and the attitudes of staff towards aggressive patient behaviour. Furthermore, the available cumulative data did not allow us to determine to which extent case-mix differences accounted for the differences between hospitals. Differences in the data between the hospitals can therefore not solely be attributed to different local or national traditions in the treatment of patients with schizophrenic disorders.

Though data on the use of coercive measures of the kind presented here is obviously highly relevant for the quality of inpatient psychiatric care, there is not much data available for comparison in the literature. In a small UK study from two decades ago, less than 5% of admissions were reported to be secluded, with forced medication not being reported [4]. An earlier study from the USA [12] reported that 1.9 % out of 5580 adult inpatients were exposed to seclusion or restraint. Review articles, based on retrospective studies of mostly small patient samples, showed that seclusion and restraint rates vary widely and that the use of these measures seemed to depend on staff rather than on patient characteristics [13-15]. All these previous studies, however, do not analyse the data regarding different diagnostic groups. In other publications indicators used for comparison were calculated differently compared to this study by using the number of measures per 100.000 inhabitants [7] (Germany) or per inpatient days [16] (USA), but not taking into account the number of admissions. This renders comparisons impossible. The data from other studies is related only to single units [17] (UK), [18] (Australia), [19] (USA) and can therefore not be compared with our data, either. Except for one study from Finland [6] there seems to be no comparable data. For admissions with schizophrenic disorders (ICD-10 F2 diagnosis) Kaltiala-Heino et al. [6] report rates of 9.3% exposed to seclusion and 5.7% of admissions exposed to mechanical restraint across three psychiatric hospitals in Finland. For seclusion this is about half that of the Swiss hospitals (17.8%) and about the same as in the German hospitals (7.8%). For mechanical restraint this is about half that of the German hospitals (10.4%) and about the same as in the Swiss hospitals (6.6%).

## Conclusion

It is not possible to determine whether the data presented here indicate better or worse clinical practice in Swiss versus German hospitals. Research is needed to analyse to what extent the use of different kinds of coercive measures, the reapplication and the duration of coercive measures are associated with psychological traumatisation of patients and the security of staff. Objectives for further research are to obtain comparable figures on the use of compulsory measures in routine psychiatric care across different countries. The working groups have helped to coordinate data collection across countries and efforts are currently being made to collect the same data in Wales. The continuous surveillance of key indicators will show possible effects of changes in health care policy, admission policy, staffing levels and treatment guidelines on the use of coercive measures in clinical practice. Beyond all methodological difficulties, comparisons across countries give an external point of reference and allow critical reflection of national traditions. They allow for knowledge transfer and help to achieve transparency on an international level. The preconditions of such international comparisons are uniformed definitions and reliable documentation of coercive measures as well as an identical way of data analysis. To meet these conditions is the first step to achieve European standards for the use of coercive measures.
